# Supplementary material for: Venomix: a simple bioinformatic pipeline for identifying and characterizing toxin gene candidates from transcriptomic data
Source: PeerJ. 2018 Jul 31;6:e5361. doi: 10.7717/peerj.5361 (PMC6074769; doi:10.7717/peerj.5361)
Supplement: Supplemental Information 6 [file peerj-06-5361-s006.gz › FinalOutput_1E-20/Zinc_metalloproteinase@disintegrin_107/finaltree.pdf]

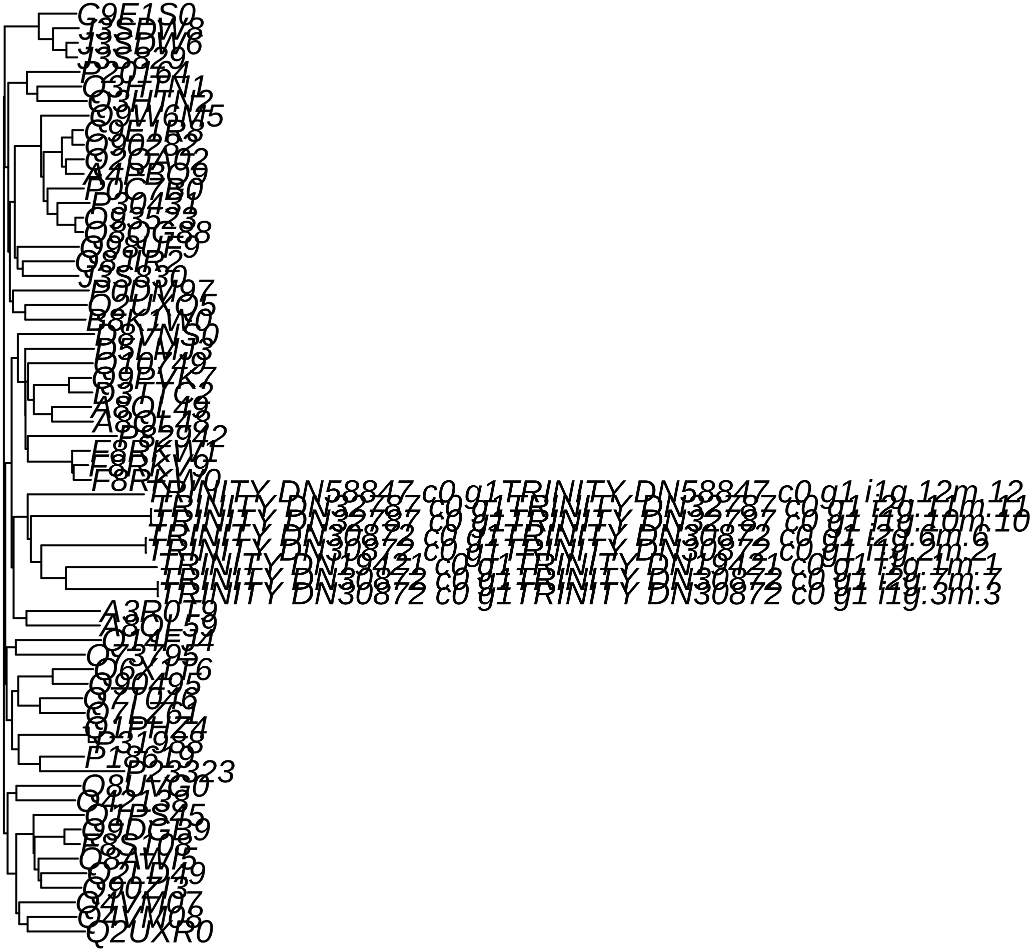
C9E1S0
Q9W6M5
B8K1W0
TRINITY DN58847 c0 g1TRINITY DN58847 c0 g1 i1g.12m.12
TRINITY DN30872 c0 g1TRINITY DN30872 c0 g1 i1g.3m.3
A3R0T9
P23323
Q8LWG0
Q2UXR0
